# Changes in Food Consumption and Eating Behaviours of Children and Their Families Living in Italy during the COVID-19 Pandemic: The EPaS-ISS Study

**DOI:** 10.3390/nu15153326

**Published:** 2023-07-26

**Authors:** Paola Nardone, Angela Spinelli, Marta Buoncristiano, Silvia Andreozzi, Mauro Bucciarelli, Marco Giustini, Silvia Ciardullo, on behalf of the EPaS-ISS Group

**Affiliations:** 1 National Centre for Disease Prevention and Health Promotion, Italian National Institute of Health, Viale Regina Elena 299, 00161 Rome, Italy; paola.nardone@iss.it (P.N.); angela.spinelli@iss.it (A.S.); silvia.andreozzi@iss.it (S.A.); mauro.bucciarelli@iss.it (M.B.); 2 “OKkio alla SALUTE” Technical Committee, 00161 Roma, Italy; marta.buoncristiano@gmail.com; 3 Environment and Health Department, Italian National Institute of Health, Viale Regina Elena 299, 00161 Rome, Italy; marco.giustini@iss.it

**Keywords:** children, family, COVID-19 pandemic, food consumption, eating behaviours

## Abstract

The aim of the EPaS-ISS study was to describe the changes in food consumption and eating behaviours of children (mainly aged 8–9 years) and their families between the pre-COVID-19 period (before February/March 2020) and the COVID-19 period (from February/March 2020 to April 2022). A web questionnaire completed by parents was used to collect data. The sociodemographic characteristics of the children and their parents were also explored through the web questionnaire. Seventeen regions out of nineteen and the two autonomous provinces (PA) of Italy participated in the study. The survey was completed for 4863 children (47.9% females). The study showed that only small changes in children’s food consumption happened between the pre-COVID-19 period and the COVID-19 period; in particular, about 25% of parents reported an increase in savoury snacks and sweet food. A decrease in fruit and vegetable (about 8%) and fish (14%) consumption was also found. However, the changes seem to have mainly affected children from most disadvantaged families. The results also indicate positive changes during the COVID-19 pandemic in some families’ eating behaviours, such as eating more home-cooked meals (42%) and family meals (39%), as well as cooking more with children (42%).

## 1. Introduction

During the past three years, the COVID-19 pandemic dominated people’s lives. On 11 March 2020, the World Health Organization (WHO) declared COVID-19 a global pandemic [1]. Due to high transmission rates of the SARS-CoV-2 virus, several governments worldwide implemented several containment measures against the disease, such as school closures, social distancing and/or quarantine at home. The Italian government enacted many restrictions concerning the movement of people and their physical distancing, including periodic lockdowns. As a result of this, family routines were interrupted, causing pronounced changes in people’s social lives and in their lifestyles.

In Italy, schools of all grades were closed in February/March 2020, and students continued their academic school programs at home until the end of the school year. Later, relaxing the levels of restrictions gradually allowed greater mobility, while maintaining the suspension of some activities depending on the situation in each Italian region and autonomous province. During this period, schools were closed to students and school personnel positive for the SARS-CoV-2 virus and distance learning was adopted.

Childhood is a critical period of life: healthy behaviours such as a balanced diet, an appropriate level of physical activity and an appropriate duration of sleep during this period are essential for both physical health and mental wellbeing and for the adequate development of young people that may persist into adulthood. Childhood is also a time of opportunity; investments and experiences during the early childhood period create the foundations for lifetime success [2].

The COVID-19 pandemic and the resulting confinement at home may have led to unfavourable responses among children that could persist after the pandemic and may have a greater impact for more socioeconomically disadvantaged children. Consequently, the sociodemographic inequality gap might have been increased during the pandemic, involving mainly vulnerable children since poor lifestyles and the difficulties for their families have already negatively affected their life [3].

Many studies have focused on the importance of assessing the influence of lockdowns and the pandemic period on children. Some authors have noticed changes in dietary habits, other lifestyle aspects and the weights of children and adolescents in Italy. Farello et al. [4] observed an increase in home-cooked meals and high-carbohydrate foods, whereas others showed an increase in snack consumption and skipping meals [5]. These changes in children’s lifestyles might have an important impact, especially on some categories, such as young people with overweight and obesity. An Italian study investigated the lifestyle variations during the lockdown period in a small number of children and adolescents with obesity, showing an increase in fruit consumption but also in unhealthy food such as salty snacks and sugar-sweetened beverages [6]. Similar findings were found in other countries. Carrol et al. (2020) reported that in many families with children in Canada, there was a large increase in both salty and sugary snack consumption [7]. In contrast, the same study showed that parents and children ate less fast food and takeaways and many parents spent more time cooking together with their children and consumed meals with the family more often. A study in Greece showed an increase in the consumption of fruit and fresh fruit juices, vegetables, dairy products, pasta, sweets, total snacks, and breakfast in adolescents [8]. A study conducted in five countries found an increased intake of legumes, fruits and vegetables but also a higher sweet food consumption in children aged 10–19 years [9]. In France, changes in child boredom and parental stress were found to influence eating and feeding behaviours and some parental characteristics were associated with feeding and cooking behaviours [10].

In Italy, a web survey was carried out in the framework of the Project EPaS-ISS “Effects of COVID-19 pandemic on health behaviour and lifestyle of children and their families living in Italy” that was promoted and funded by the Italian National Institute of Health (Istituto Superiore di Sanità, ISS, Rome, Italy) and coordinated by its National Centre for Disease Prevention and Health Promotion (Centro Nazionale per la Prevenzione delle Malattie e la Promozione della Salute, CNaPPS, Rome, Italy). The study is part of the WHO European Childhood Obesity Surveillance Initiative (COSI) and conducted in 13 countries [11].

This article presents the results of the study on the changes in food consumption and eating behaviours of children and their families in Italy between the pre-pandemic period (before February/March 2020) and the pandemic period (from February/March 2020 to April 2022 based on the periods of suspension of face-to-face school activities).

## 2. Materials and Methods

A web questionnaire was used to collect data. The target population was the parents of children attending the third grade of primary schools (mainly aged 8 and 9 years). Data were collected according to a common protocol. Following the COSI sampling approach, a two-stage stratified cluster sample design was used, with schools as the primary sampling units and classes as the secondary sampling units [11,12]. All parents of the selected classes (two or three for each school) were invited to participate. The selection of primary sampling units was from the primary schools previously involved in the 2019 survey of “OKkio alla SALUTE”, which is part of the COSI [13,14]. Seventeen regions out of nineteen and the two autonomous provinces (PA) of Italy participated in the study. Regional and PA coordinators, in collaboration with local health unit (LHU) coordinators, carried out and supported the activities at the local level. The EPaS-ISS study was presented to schools by regional and LHU coordinators with the support of provincial school offices (PSO). The parents of the enrolled classes received information about the study, as well as the link to access the web questionnaire, from head teachers and teachers.

Data collection began in early April 2022 and finished at the end of September 2022. The web questionnaire for parents was developed using LimeSurvey software (version 6.1.7) and optimised according to the type of device used for its compilation (i.e., smartphone, tablet or PC). An information note with the description of the aim of the study and the consent to participate, the privacy policy for participation in the study and the consent to the processing of personal data was also implemented online. The questionnaire, information note and privacy policy were available online in Italian, English, Arabic and Chinese.

Parents were asked if their children’s consumption of some foods (fresh fruit, vegetables, meat, fish, dairy products, pulses and legumes, savoury snacks, sweet food, packaged drinks containing sugar and breakfast cereals) decreased, stayed the same or increased during the pandemic period (COVID-19 period = from February/March 2020 to April 2022 based on the periods of suspension of face-to-face school activities) in comparison with the pre-COVID-19 period (before February/March 2020). The study also investigated how the pandemic affected some family eating behaviours such as buying food online, buying food in bulk (over a period longer than a week), eating home-cooked meals, eating food prepared outside the home (e.g., takeaway food ordered online or prepared by a restaurant), eating together as a family and cooking with children. Parents were asked how these consumption behaviours have changed during the pandemic period compared with the pre-COVID-19 period. The sociodemographic characteristics of parents were also collected through the web questionnaire. The sociodemographic characteristics included in the study were: children’s gender (males, females), families’ area of residence (north, centre, south), parents’ nationality (Italian, foreign; categorised as: both Italian, at least one foreign parent), parents’ educational level (primary school, secondary school, high school, degree, master/doctorate/specialisation; in the analyses the highest level between the two parents was used to classify them as follows: low = both parents with less than high school, medium = at least one of the parents with high school, high = at least one of the parents with a university degree or higher) and family structure (two-parent family, single-parent family). Items from the questionnaire regarding changes in the children’s food consumption and families’ eating behaviours are reported in Table 1. A list of each question considered in the analysis is shown in Supplementary Material S3. Frequency distributions by sociodemographic characteristics were estimated and the prevalence of the outcome variables was stratified. Differences in the prevalence according to the socio-demographic characteristics were tested by the Pearson-design-based χ^2^.

Logistic regression models were used to explore the association between gender, geographic area, parents’ nationality and educational level, as well as family structure (independent variables) and changes in children’s food consumption and families’ eating behaviours (dependent variables), taking into account for each response variable the increase vs. stayed the same or decreased (yes/no) or decreased vs. stayed the same or increased (yes/no) during the COVID-19 pandemic period. The likelihood was described by odds ratios (OR) with their 95% confidence intervals (CI). Missing data and “Don’t know” responses were excluded from the analysis.

Weights to adjust for oversampling and nonresponse were used. All analyses took into account the clustered and stratified nature of the data and used STATA (Stata Statistical Software: v. 15, Release 15, College Station, TX, USA: Stata-Corp LP). A *p*-value of 0.05 or less was considered as statistically significant.

## 3. Results

### 3.1. Characteristics of Children and Parents

Around 5900 parents gave their consent to participate in the survey, equal to 46.6% of families that were invited. Children with parents that answered only the initial few questions were excluded from the analysis. A total of 4863 children were included: 47.9% were females, the average age was 8 years and 9 months (SD ± 5 months) and they were distributed as follows: north: 47.5%; centre: 24.4%; south: 28.1%. The percentage of parents with missing responses ranged from 0% to 1.5%, with the exceptions of questions about parents’ educational level (mother educational level: 6.9%; father educational level: 8.1%), parents’ nationality (mother nationality: 6.6%; father nationality: 6.8%) and families’ consumption behaviours, i.e., buying food online (4.2%) and eating food prepared outside the home (2.4%). The characteristics of the parents are reported in Table 2. The majority of parents were Italian. Taking into account the highest level of education between mother and father, most of the parents reported having a medium or high educational level.

### 3.2. Change in Children’s Food Consumption

The results concerning changes in children’s food consumption between the pre-COVID-19 period and the COVID-19 period for the total sample are shown in Table 3. Table S1 (Supplementary Material) shows these changes stratified by children’s gender, geographical area of residence and the parents’ nationality and educational level, as well as the family structure.

Overall, about 70–80% of the 4863 parents who participated in the survey claimed no changes in the consumption of foods selected for the study. However, 23.9% of parents reported an increase in the intake of savoury snacks and sweet food (25.3%) but a decrease in the consumption in fish (13.8%) among their children. After stratification, the reported changes in children’s food consumption were associated with gender, geographical area, parents’ nationality and educational level and family structure. 

If a change in the consumption of fruit and vegetables was reported, it tended to be more of a decrease (fresh fruit: 8.0%; vegetables: 8.9%) rather than an increase in the COVID-19 period compared with the pre-COVID-19 period. Data stratified for the sociodemographic characteristics of children and parents showed a greater decrease in the consumption of fresh fruit among children who lived in the southern regions (north: 7.2%; centre: 7.8%; south: 9.2%), those with at least one parent with a medium or high level of education (low: 4.7%; medium: 8.2%; high: 8.2%), those with at least one foreign parent (two Italian parents: 7.9%; at least one foreign parent: 9.02%) and those living in a single-parent family (two-parent family: 8.0%; single-parent family: 8.8%). With regard to vegetables, a major intake decrease was reported among children with at least one parent with a medium or high educational level (low: 7.1%; medium: 10.0%; high: 8.1%), with at least one foreign parent (two Italian parents: 8.8%; at least one foreign parent: 10.1%) and among children who lived in a two-parent family (two-parent family: 9.2%; single-parent family: 7.7%).

The consumption of meat, dairy products, pulses and legumes, packaged drinks containing sugar and breakfast cereals showed very little change between the pre-COVID-19 period and the pandemic period, with similar percentages in the reported increased and the decreased intakes.

Seven percent of parents claimed a decrease in meat consumption among children; a comparable percentage of parents (7.23%) also declared an increase in the consumption of meat during the pandemic.

The percentage who reported a decrease in fish consumption (13.8%) increased from the north to the south (north: 12.4%; centre: 13.5%; south: 15.9%). Moreover, a major decline in fish intake was also found among children with at least one parent with a medium or high educational level (low: 11.7%; medium: 14.2%; high: 14.2%,) and those who lived with parents that were both Italian (two Italian parents: 14.7%; at least one foreign parent: 7.3%).

Our results revealed that some children increased their consumption of savoury snacks and sweet foods during the COVID-19 pandemic, with differences depending on sociodemographic characteristics. In detail, the stratified data showed that children from centre and south of Italy mainly increased the frequency of savoury snack consumption in comparison with those from northern regions (north: 21.9%; centre: 25.2%; south: 25.7%); a major increase was observed for children with at least one parent with a medium or high educational level (low: 20.7%; medium: 26.4%; high: 22.7) and among children who lived with two Italian parents (two Italian parents: 24.3%; at least one foreign parent: 23.3%). For sweet food consumption, the stratified results were comparable with savoury snacks, and significant differences were found for geographical areas (north: 23.6%; centre: 26.1%; south: 27.2%), parents’ educational level (low: 21.7%; medium: 26.4%; high: 25.9%) and nationality (two Italians parents: 26.3%; at least one foreign parent: 21.5%).

### 3.3. Change in Families’ Eating Behaviours

Table 4 shows the changes in families’ eating behaviours between the pre-COVID-19 period and the pandemic period for the total sample. The results regarding the changes in families’ eating behaviours stratified by gender, geographical areas, parents’ nationality and educational level, as well as family structure, are reported in Table S2 (Supplementary Material).

Our findings indicate that some the eating behaviours of families resident in Italy changed during the COVID-19 pandemic. In addition, a strong association between some eating behaviours and the sociodemographic characteristics of children and their families was observed.

Buying food online increased in 18.9% of families but decreased in 16.9%; 23.2% replied that they did not know (Table 4). The differences in the increased buying of food online were observed by gender, geographical area and parents’ nationality and educational level. An increasing trend was observed from south to north in terms of geographical area (from 11.6% to 25.0%) and from low to high in terms of parents’ educational level (from 5.96% to 24.0%). Families where both parents were Italian showed a greater increase in buying food online compared with families with at least one foreign parent (two Italian parents: 20.3%; at least one foreign parent: 10.2%). However, marked differences in parents’ “Don’t know” response related to geographical area, educational level and nationality were also observed (Table S2), with higher percentages for parents from southern regions (29.9%) and with a low educational level (40.4%), as well as families with at least one foreign parent (35.1%).

Almost half of parents (47.1%, Table 4) reported buying more food in bulk (over a period longer than a week). A marked trend, increasing from low to high in terms of the parents’ level of education was observed (low: 31.6%; medium: 45.1%; high: 53.2%, Table S2). Significant differences were also found stratifying for parents’ nationality and the family structure; a greater increase was observed for families with two Italian parents (two Italian parents, 48.8%; at least one foreign parent, 37.5%) and with two-parents (two parent family, 49.3%; single-parent family 40.2%).

A total of 42.2% of parents claimed to eat more home-cooked meals (Table 4) and a marked increasing trend from low (31.5%) to high (46.2%) was observed for the level of education of the parents (Table S2). A higher increase was also revealed for families with two Italian parents (two Italian parents, 44.2% vs. at least one foreign parent, 29.7%) and with two parents (two-parent family, 43.7% vs. single-parent family 38.5%).

The results show a decrease in eating food prepared outside the home during the COVID-19 pandemic period (28.1%, Table 4). Stratifying by geographical area, a major decrease was observed in the southern regions (Table S2). Furthermore, parents with a low level of education and families with at least one foreign parent claimed to have had a greater decrease in eating food prepared outside the home than those with a medium or high level of education and with two Italian parents, respectively. However, 12.8% of families reported an increase and 15.1% did not know. “Don’t know” responses increased for parents from southern regions (20.0%, with low educational level (28.1%) and families with at least one foreign parent (21.1%, Table S2).

Our findings also show a high percentage of parents who declared an increase in eating together as a family (39.2%) and cooking with children (42.2%) (Table 4); for both eating behaviours, a major increase was ascertained for parents with higher educational levels as well as for families with parents who were both Italian and with two parents. Parents from Northern and Central Italy claimed a greater increase in eating together as a family than those from the Southern Italy (Table S2).

### 3.4. Multivariate Analysis

The results of the logistic regression models on changes in children’s food consumption and families’ eating behaviours are reported in Table 5 and Table 6, respectively. They do not contradict the results of the univariate analyses, although in some cases the adjusted odds ratios are not statistically significant.

In detail, statistically significant odds ratios for decreases in the intakes of fruit (OR: 1.53; 95% CI: 1.17–1.99), vegetables (OR: 1.34; 95% CI: 1.04–2.73) and fish (OR: 1.37; 95% CI: 1.11–1.70) were observed among children from southern regions in comparison with the children living in northern regions (Table 5), as well as an increase in the intake of savoury snacks (OR: 1.23; 95% CI: 1.03–1.46). For savoury snack consumption, a statistically significant odds ratio increase was also found for children resident in the central regions of Italy (OR: 1.25; 95% CI: 1.04–1.49). Furthermore, a higher decrease in fruit and vegetable consumption was associated with the parents’ educational level (medium and high educational level). Gender differences were observed for the decrease in meat and fish consumption; in both cases, the decrease was significantly higher among girls than boys. Children with at least one foreign parent showed a higher risk for decreasing meat consumption (OR: 1.47; 95% CI 1.03–2.09) but a lower risk for decreasing fish consumption in comparison with children with two Italian parents (OR: 0.54; 95% CI: 0.37–0.79). None of the independent variables showed statistically significant adjusted odds ratios for the increase in sweet food consumption.

Regarding the eating behaviours in the family weekly routine, families with at least one parent with a medium or high educational level were more likely to have increases in buying food online (medium, OR: 2.27; 95% CI: 1.38–3.72; high, OR: 2.84; 95% CI: 1.73–4.66), buying food in bulk (medium, OR: 1.63; 95% CI: 1.26–2.12; high, OR: 2.06; 95% CI: 1.58–2.69), eating home-cooked meals (medium, OR: 1.48; 95% CI: 1.14–1.93; high, OR: 1.71; 95% CI: 1.31–2.23) and eating together as a family (medium, OR: 1.87; 95% CI: 1.40–2.49; high, OR: 2.55; 95% CI: 1.91–3.39), as well as cooking with children (medium, OR: 1.46; 95% CI: 1.11–1.91; high, OR: 1.83; 95% CI: 1.40–2.39). A high parent education level was associated with a minor decrease in eating food prepared outside the home (OR: 0.61; 95% CI: 0.46–0.82). As shown in the univariate analyses, the increase in buying food online during the COVID-19 period was smaller in the centre (OR: 0.73; 95% CI: 0.59–0.89) and south of Italy (OR: 0.46; 95% CI: 0.37–0.58). Living in the south of Italy also showed a statistically significant effect on the decrease in eating food prepared outside at home and on the increase in eating together as a family. The nationality of the parents had an important effect on all changes in the families’ consumption behaviour, with bigger changes in families where both parents were Italian.

## 4. Discussion

The aim of this study was to investigate the changes in food consumption and eating behaviours of children and their families during the COVID-19 pandemic. The EPaS-ISS study considered not only the national lockdown but each period in which children were mostly prevented from attending school (e.g., resident in an Italian “red” zone, being in self-isolation or because the school attended was closed). This approach was adopted in the framework of the COSI study and allowed us to compare the results with the other involved countries [11].

Our study showed that only small changes in children’s food consumption happened between the pre-COVID-19 period (before February/March 2020) and the COVID-19 period (from February/March 2020 to April 2022); in particular, about 25% of parents reported an increase in savoury snack and sweet food consumption and about an 8% increase in fruit and vegetable consumption. Before the COVID-19 pandemic, the consumption of these foods among children living in Italy was already not optimal. Data from the sixth round of the surveillance system “OKkio alla SALUTE”, which was carried out in 2019, found that 24% of 8–9-year-old Italian children did not consume fruits and vegetables daily [13]. It also showed that 9.4% of children consumed savoury snacks on at least four days during the week and 2.9% consumed them every day; a major intake was found for sweet snacks, which were consumed on at least four days during the week and every day for 48.4% and 19.9% of children, respectively. 

It is well known that diets rich in fruits and vegetables have health-protective effects and may help to prevent overweight and obesity in children [15,16]. Even though the WHO recommends that people eat a variety of fruits and vegetables five or more times a day [15], the majority of children and adolescents in Italy do not meet these dietary guidelines [13,17,18]. Another study found a decrease in fruit and vegetable intake during the pandemic period [19], whereas Palermi et al. (2022) showed no change in the consumption of fruits and vegetables among Italian children and adolescents aged 8–15 years [5]. An increase in the intake of fruits and vegetables during the COVID-19 pandemic was observed by a Polish study on primary school students aged 10–16 years [20]. Comparable results were reported in the study by Androutsos et al. (2021), which showed an increase in fruit and vegetable consumption in the diets of 2–18-year-old Greek children [8]. Our study found higher percentages for a decrease in the consumption of fruits and vegetables among children with lower educated parents and living in the south of Italy, the poorest part of the country. Similar results were reported by Censi et al. (2022) [19].

Due to containment measures against the COVID-19 disease, children spent more time at home indoors and the change in their regular routines might have resulted in an increase in eating meals and snacks during the day. Snacking may contribute to energy-dense and nutrient-poor diets; high rates of snacking as well as snacks of generous portion sizes have been associated with increased energy intake and weight gain [15,21,22]. There is evidence that most snacks consumed by children are highly processed and high in salt, fat or sugar, such as confectioneries and crisps [23]. The increase in the consumption of savoury snacks and sweet foods during the COVID-19 period shown in our EPaS-ISS investigation, especially in the centre and south of Italy, was found in other studies. Censi et al. (2022) showed a marked increasing north–south trend in the percentage of children who consumed sweets and candies [19]. Palermi et al. (2022) reported that Italian children and adolescents aged 8–15 years ate more junk food and snacks [5]. A greater consumption of desserts and high-carbohydrate foods among Italian children during lockdown was ascertained by Farello et al. (2022) [4]. At the international level, a study conducted in Western Sydney (Australia) on changes in the intake of some foods by children and their families during the COVID-19 pandemic highlighted an increase in eating meals and snacks [24]. Some authors have noted the relationship between not attending school during holidays and an increase in some unhealthy behaviours among young people, such as an inadequate diet; due to home confinement during the pandemic, a similar behaviour among children was observed [25]. Altogether, the results regarding the increase in snacking during the pandemic support the importance of deeply investigating this behaviour among children to gain knowledge of the determinants that might affect this behaviour during childhood [26,27,28] and to promote the correct daily consumption of snacks among children.

Our results on the eating behaviours of children and their families revealed several changes during the COVID-19 period, highlighting that the pandemic deeply influenced people’s lifestyles. Some eating behaviours in a family’s weekly routine, e.g., buying food online, buying food in bulk, eating home-cooked meals, eating food prepared outside the home, eating together as a family and cooking with children, showed pronounced differences between the pre-COVID-19 period and the COVID-19 period, and these differences were associated with nationality and educational level of parents.

As previously reported by other authors, families had some difficulties in finding certain foodstuffs during the pandemic (e.g., yeast, flour, fish, fruit and vegetables) [19]; together with the uncertainty about the future, this might have influenced the increase in buying food in bulk that was revealed by our results. Some authors also reported changes in the total amount of food in the home, confirming the hoarding by families during the pandemic period [4]. There were also positive eating behaviour changes during the COVID-19 pandemic. The parents involved in our investigation claimed to eat more home-cooked meals and less food prepared outside the home. Censi et al. (2022) ascribed these changes to the preparation of leavened food products [19]. Furthermore, many parents spent more time at home and the opportunity of smart working allowed their involvement in domestic activities. Our findings highlighted that children consumed more meals together as a family and increased the habit of cooking with their parents; this was in accordance with other studies [5,19,24]. Altogether, the changes in families’ eating behaviours, such as eating more home-cooked meals, eating more meals together and cooking with children, were positively associated with parents having a high or medium educational level and being Italian.

### Strengths and Limitations

The main strengths of the EPaS-ISS study were the use of standardised data collection procedures based on the international COSI, as well as the support of regional coordinators and local health unit personnel involved in the “OKkio alla SALUTE” surveillance system. The use of a large Italian sample from almost all Italian regions to investigate the impact of the COVID-19 pandemic on children’s food consumption and some eating behaviours of families is another strength of this study. However, EPaS-ISS also had some limitations. The first is the possibility of selection bias due to low response rate. Data on the educational level obtained from the EPaS-ISS study were compared with those from the “OKkio alla SALUTE” surveillance system, which, in 2019, achieved a response rate of 95%. The comparison found a smaller percentage of parents with a lower level of education in the EPaS-ISS study compared with the 2019 “OKkio alla SALUTE” survey. Being an Internet user, i.e., having basic technical skills such as being able to use computers, tablets or smartphones, was required to take part in the investigation and could have been a source of selection bias. It is possible that more vulnerable families were less likely to participate in this study; therefore, this may have led to lower representation from these groups or even an underestimation of inequalities. Another issue is related to the high number of “Don’t know” responses for some questions on families’ eating behaviours such as changes in buying food online and eating food prepared outside the home. These questions were probably not well understood by the parents.

## 5. Conclusions

The EPaS-ISS study has shown very little change in food consumption among children living in Italy during the COVID-19 pandemic (thanks to the great efforts of the Italian population), except for an increase in the consumption savoury snacks and sweet food and a little decrease in the consumption of fruits and vegetables. However, the changes seem to mainly involve children from the most disadvantaged families. The results from the EPaS-ISS study also revealed positive changes during the COVID-19 pandemic in some families’ consumption behaviours, such as eating more home-cooked meals and family meals, as well as cooking more with children. This was possibly due to spending more time together. It will be interesting to monitor if these changes remain and have an effect on nutritional status of children by using a routine surveillance system such as the Italian OKkio alla SALUTE survey. Moreover, further research should review the policies and strategies adopted by other countries to limit unhealthy behaviours and to promote positive change in the lifestyles of children and their families. Particular attention should be paid to the most disadvantaged families, where unhealthy habits are already more common and who might need special interventions.

## Figures and Tables

**Table 1:** Items from questionnaire about changes in children’s food consumption and families’ eating behaviours.

Question	Response
**Children’s Food Consumption**	
Please indicate whether there were any changes in your child’s consumption of the following foods during the COVID-19 period compared to the PRE-COVID period 1. Fresh fruit 2. Vegetables (including soups and pureed vegetables but excluding potatoes) 3. Meat 4. Fish 5. Dairy products (e.g., milk, yoghurt, cheese) and eggs 6. Pulses and legumes 7. Savoury snacks (e.g., crisps, popcorn, nuts, crackers) 8. Sweet foods (e.g., cakes, snacks, biscuits, sweets, ice cream) 9. Packaged drinks containing sugar, (e.g., tea, orange soda, cola, fruit juices) 10. Breakfast cereals (e.g., corn flakes, muesli)	Decreased, Stayed the same, Increased, Don’t Know
**Families’ eating behaviours**	
Please indicate which of the following behaviours in a normal week during the COVID-19 period were different from the PRE-COVID period: 1. Buying food online 2. Buying food in bulk (over a period longer than a week) 3. Eating home-cooked meals 4. Eating food prepared outside the home (e.g., takeaway food ordered online or prepared by a restaurant) 5. Eating together as a family 6. Cooking with your child	Decreased, Stayed the same, Increased, Don’t Know

**Table 2:** General characteristics of parents.

Sociodemographic Variables		%	IC 95%
**Mother educational level**			
	*None*	0.4%	0.25–0.66
	*Primary school (6 to 11 years)*	0.5%	0.31–0.74
	*Secondary school (from 11 to 14 years)*	12.0%	11.09–13.01
	*High school (from 14 to 19 years)*	46.9%	45.44–48.38
	*Degree*	31.8%	30.43–33.18
	*Master/Doctorate/Specialisation*	8.4%	7.57–9.21
**Mother nationality**			
	Italian	90.0%	89.12–90.89
	Foreign	9.8%	8.91–10.67
**Mother not present**		0.2%	0.09–0.38
**Father educational level**			
	*None*	0.5%	0.31–0.75
	*Primary school (6 to 11 years)*	1.2%	0.88–1.53
	*Secondary school (from 11 to 14 years)*	22.2%	20.98–23.45
	*High school (from 14 to 19 years)*	50.4%	48.90–51.86
	*Degree*	21.0%	19.77–22.19
	*Master/Doctorate/Specialisation*	4.8%	4.19–5.47
**Father nationality**			
	Italian	91.5%	90.65–92.30
	Foreign	7.3%	6.60-8.15
**Father not present**		1.1%	0.09–1.51

**Table 3:** Change in children’s food consumption between the pre-COVID-19 period and the COVID-19 period.

Children’s Eating Habits				
	Decreased (%, 95% CI)	Stayed the Same (%, 95% CI)	Increased (%, 95% CI)	Don’t Know (%, 95% CI)
**Fresh fruit**	8.0 (7.21–8.92)	80.0 (78.6–81.2)	6.9 (6.23–7.66)	5.2 (4.51–5.89)
**Vegetables (including soups and pureed vegetables but excluding potatoes)**	8.9 (8.04–9.91)	80.8 (79.6–82.0)	5.8 (5.13–6.52)	4.5 (3.88–5.13)
**Meat**	7.4 (6.58–8.21)	82.0 (80.8–83.1)	7.2 (6.53–7.99)	3.4 (2.93–4.01)
**Fish**	13.8 (12.8–15.0)	77.5 (76.1–78.8)	3.9 (3.3–4.53)	4.8 (4.21–5.44)
**Dairy products (e.g., milk, yoghurt, cheese) and eggs**	5.6 (4.94–6.38)	82.7 (81.4–83.9)	8.0 (7.23–8.87)	3.7 (3.14–4.35)
**Pulses and legumes**	6.4 (5.71–7.08)	82.6 (81.6–83.6)	5.1 (4.47–5.90)	5.9 (5.26–6.63)
**Savoury snacks (e.g., crisps, popcorn, nuts, crackers)**	5.9 (5.18–6.69)	66.8 (65.1–68.4)	23.9 (22.4–25.4)	3.5 (2.97–4.05)
**Sweet foods (e.g., cakes, snacks, biscuits, sweets, ice cream)**	5.1 (4.47–5.84)	66.2 (64.6–67.7)	25.3 (23.9–26.9)	3.4 (2.85–4.00)
**Packaged drinks containing sugar, (e.g., tea, orange soda, cola, fruit juices)**	9.8 (8.99–10.7)	75.1 (73.7–76.5)	10.6 (9.58–11.7)	4.4 (3.85–5.04)
**Breakfast cereals (e.g., corn flakes, muesli)**	5.7 (5.03–6.46)	81.0 (79.8–82.1)	6.1 (5.53–6.83)	7.1 (6.35–8.03)

**Table 4:** Change in children’s eating behaviors between the pre-COVID-19 period and the COVID-19 period.

Families’ Consumption Behaviours	Decreased (%, IC 95%)	Stayed the Same (%, IC 95%)	Increased (%, IC 95%)	Don’t Know (%, IC 95%)
**Buying food online**	16.9 (15.7–18.1)	41.1 (39.4–42.8)	18.9 (17.3–20.6)	23.2 (21.7–24.7)
**Buying food in bulk (over a period longer than a week)**	6.6 (5.93–7.39)	40.4 (38.8–42.1)	47.1 (45.3–49)	5.8 (5.1–6.63)
**Eating home-cooked meals**	2.7 (2.21–3.31)	52.2 (50.6–53.8)	42.2 (40.5–43.8)	2.9 (2.4–3.51)
**Eating food prepared outside the home (e.g., takeaway food ordered online or prepared by a restaurant)**	28.1 (26.7–29.6)	43.9 (42.3–45.6)	12.8 (11.8–14)	15.1 (13.8–16.4)
**Eating together as a family**	7.7 (6.71–8.74)	50.7 (48.9–52.4)	39.2 (37.3–41)	2.5 (2.04–3.05)
**Cooking with your child**	3.8 (3.21–4.42)	50.0 (48.3–51.6)	42.0 (40.2–44)	4.2 (3.64–4.92)

**Table 5:** Logistic regression models for changes in children’s food consumption between the pre-COVID-19 period and the COVID-19 period *.

Indipendent Variables	Fruit Consumption Decreased (Yes/No)	Vegetables Consumption Decreased (Yes/No)	Meat Consumption Decreased (Yes/No)	Fish Consumption Decreased (Yes/No)	Savoury Snack Consumption Increased (Yes/No)	Sweet Consumption Increased (Yes/No)
	OR ** (95% CI)	OR ** (95% CI)	OR ** (95% CI)	OR ** (95% CI)	OR ** (95% CI)	OR ** (95% CI)
**Gender**						
Males	1	1	1	1	1	1
Females	0.93 (0.74–1.16)	1.06 (0.85–1.32)	**1.50 (1.18–1.90)**	**1.20 (1.00–1.44)**	0.91 (0.79–1.05)	1.00 (0.87–1.15)
**Residence area**						
North	1	1	1	1	1	1
Centre	1.17 (0.87–1.56)	0.92 (069–1.22)	0.91 (0.68–1.22)	1.17 (0.94–1.47)	**1.25 (1.04–1.49)**	1.15 (0.96–1.37)
South	**1.53 (1.17–1.99)**	**1.34 (1.04–2.73)**	0.79 (0.59–1.07)	**1.37 (1.11–1.70)**	**1.23 (1.03–1.46)**	1.13 (0.95–1.34)
**Parents’ nationality**						
Both Italians	1	1	1	1	1	1
At least one foreign parent	1.33 (0.93–1.90)	1.37 (0.97–1.92)	**1.47 (1.03–2.09)**	**0.54 (0.37–0.79)**	1.01 (0.80–1.29)	0.84 (0.66–1.08)
Parent’s educational level ***						
Low level of education	1	1	1	1	1	1
Medium level of education	**1.73 (1.01–2.94)**	1.59 (0.98–2.57)	1.31 (0.79–2.18)	1.15 (0.79–1.66)	**1.34 (1.00–1.81)**	1.19 (0.89–1.59)
High level of education	**1.71 (1.00–2.92)**	1.24 (0.76–2.02)	1.10 (0.65–1.84)	1.09 (0.75–1.59)	1.09 (0.81–1.47)	1.12 (0.83–1.50)
**Family structure**						
Two-parent family	1	1	1	1	1	1
One-parent family	1.18 (0.87–1.60)	0.89 (0.65–1.22)	0.90 (0.65–1.25)	0.91 (0.70–1.17)	1.07 (0.87–1.30)	1.04 (0.85–1.27)

* Statistically significant results are in bold. ** Adjusted odds ratio for all variables listed in Table 5. *** The highest educational level between the two parents.

**Table 6:** Logistic regression models for changes in families’ eating behaviours between the pre-COVID-19 period and the COVID-19 period *.

Indipendent Variables	Buying Food Online Increased (Yes/No)	Buying Food in Bulk Increased (Yes/No)	Eating Home-Cooked Meals Increased (Yes/No)	Eating Food Prepared Outside the Home Decreased (Yes/No)	Eating Together as a Family Increased (Yes/No)	Cooking with Your Child Increased (Yes/No)
	OR ** (95% CI)	OR ** (95% CI)	OR ** (95% CI)	OR ** (95% CI)	OR ** (95% CI)	OR ** (95% CI)
**Gender**						
Males	1	1	1	1	1	1
Females	0.88 (0.75–1.04)	0.90 (0.79–1.02)	**0.87 (0.77–0.99)**	0.91 (0.78–1.05)	**0.88 (0.77–1.00)**	0.99 (0.87–1.13)
**Residence area**						
North	1	1	1	1	1	1
Centre	**0.73 (0.59–0.89)**	1.07 (0.91–1.25)	1.13 (0.97–1.32)	1.14 (0.95–1.37)	0.97 (0.82–1.13)	0.98 (0.84–1.15)
South	**0.46 (0.37–0.58)**	1.08 (0.92–1.26)	1.13 (0.97–1.13)	**1.99 (1.67–2.36)**	**0.72 (0.62–0.84)**	1.01 (0.87–1.18)
**Parents’ nationality**						
Both Italians	1	1	1	1	1	1
At least one foreign parent	**0.46 (0.32–0.66)**	**0.73 (0.59–0.90)**	**0.63 (0.50–0.78)**	**1.70 (1.34–2.15)**	**0.76 (0.61–0.94)**	**0.62 (0.49–0.77)**
Parent’s educational level ***						
Low level of education	1	1	1	1	1	1
Medium level of education	**2.27 (1.38–3.72)**	**1.63 (1.26–2.12)**	**1.48 (1.14–1.93)**	0.86 (0.64–1.15)	**1.87 (1.40–2.49)**	**1.46 (1.11–1.91)**
High level of education	**2.84 (1.73–4.66)**	**2.06 (1.58–2.69)**	**1.71 (1.31–2.23)**	**0.61 (0.46–0.82)**	**2.55 (1.91–3.39)**	**1.83 (1.40–2.39)**
**Family structure**						
Two-parent family	1	1	1	1	1	1
One-parent family	0.82 (0.64–1.05)	**0.70 (0.58–0.84)**	**0.81 (0.67–0.97)**	1.01 (0.82–1.24)	**0.66 (0.55–0.80)**	0.84 (0.70–1.01)

* Statistically significant results are in bold. ** Adjusted odds ratio for all variables listed in Table 6. *** The highest educational level between the two parents.

## Data Availability

The data presented in this study are available in accordance with the ISS data access policy. Requests should be directed to silvia.ciardullo@iss.it, member of the National Centre for Disease Prevention and Health Promotion, ISS.

## Supplementary Materials

The following supporting information can be downloaded at: https://www.mdpi.com/article/10.3390/nu15153326/s1, Table S1: Change in children’s food consumption between the pre-COVID-19 period and the COVID-19 period by the sociodemographic characteristics of children and parents.; Table S2: Change in families’ eating behaviours between the pre-COVID-19 period and the COVID-19 period by the sociodemographic characteristics of children and parents; S3: List of the questions considered in the analysis.

## References

[1] WHO Director-General’s Opening Remarks at the Media Briefing on COVID-19-11 March 2020. https://www.who.int/director-general/speeches/detail/who-director-general-s-opening-remarks-at-the-media-briefing-on-covid-19.

[2] Clark H., Coll-Seck A.M., Banerjee A., Peterson S., Dalglish S.L., Ameratunga S., Balabanova D., Bhan M.K., Bhutta Z.A., Borrazzo J. (2020). A future for the world’s children? A WHO-UNICEF-Lancet Commission. Lancet.

[3] (2021). COVID-19: the intersection of education and health. Lancet.

[4] Farello G., D’andrea M., Quarta A., Grossi A., Pompili D., Altobelli E., Stagi S., Balsano C. (2022). Children and Adolescents Dietary Habits and Lifestyle Changes during COVID-19 Lockdown in Italy. Nutrients.

[5] Palermi S., Vecchiato M., Pennella S., Marasca A., Spinelli A., De Luca M., De Martino L., Fernando F., Sirico F., Biffi A. (2022). The Impact of the COVID-19 Pandemic on Childhood Obesity and Lifestyle—A Report from Italy. Pediatr. Rep..

[6] Pietrobelli A., Pecoraro L., Ferruzzi A., Heo M., Faith M., Zoller T., Antoniazzi F., Piacentini G., Feambach S.N., Heymsfield S.B. (2020). Effects of COVID-19 Lockdown on Lifestyle Behaviors in Children with Obesity Living in Verona, Italy: A Longitudinal Study. Obesity.

[7] Carroll N., Sadowski A., Laila A., Hruska V., Nixon M., Ma D.W.L., Haines J., on behalf of the Guelph Family Health Study (2020). The Impact of COVID-19 on Health Behavior, Stress, Financial and Food Security among Middle to High Income Ca-nadian Families with Young Children. Nutrients.

[8] Androutsos O., Perperidi M., Georgiou C., Chouliaras G. (2021). Lifestyle Changes and Determinants of Children’s and Adolescents’ Body Weight Increase during the First COVID-19 Lockdown in Greece: The COV-EAT Study. Nutrients.

[9] Ruiz-Roso M.B., de Carvalho Padilha P., Mantilla-Escalante D.C., Ulloa N., Brun P., Acevedo-Correa D., Arantes Ferreira Peres W., Martorell M., Aires M.T., de Oliveira Cardoso L. (2020). COVID-19 Confinement and Changes of Adolescent’s Di-etary Trends in Italy, Spain, Chile, Colombia and Brazil. Nutrients.

[10] Philippe K., Chabanet C., Issanchou S., Monnery-Patris S. (2021). Child eating behaviors, parental feeding practices and food shop-ping motivations during the COVID-19 lockdown in France: (How) did they change?. Appetite.

[11] World Health Organization (WHO) Regional Office for Europe. Nutrition, Physical Activity, Well-Being and COVID-19-Results from 13 Countries Participating in Round 6 of the Childhood Obesity Surveillance Initiative Study. Technical Document 2023. https://www.who.int/europe/publications/m/item/nutrition-physical-activity-well-being-and-covid-19-childhood-obesity-surveillance-initiative-study..

[12] Breda J., McColl K., Buoncristiano M., Williams J., Abdrakhmanova Z., Ahrens W., Akhmedova D., Bakacs M., Boer J.M.A., Boymatova K. (2021). Methodology and im-plementation of the WHO childhood obesity surveillance initiative (COSI). Obes. Rev..

[13] Nardone P., Spinelli A., Ciardullo S., Salvatore M.A., Andreozzi S., Galeone D. (2022). Obesità e Stili di Vita dei Bambini: OKkio Alla SALUTE 2019.

[14] (2022). Report on the Fifth Round of Data Collection, 2018–2020: WHO European Childhood Obesity Surveillance Initiative (COSI).

[15] World Health Organization (WHO) (2014). Regional Office for Europe. European Food and Nutrition Action Plan 2015–2020.

[16] Pala V., Lissner L., Hebestreit A., Lanfer A., Sieri S., Siani A., Huybrechts I., Kambek L., Molnar D., Tornaritis M. (2013). Dietary patterns and longitudinal change in body mass in European children: A follow-up study on the IDEFICS multicenter cohort. Eur. J. Clin. Nutr..

[17] Lazzeri G., Pammolli A., Azzolini E., Simi R., Meoni V., de Wet D.R., Giacchi M.V. (2013). Association between fruits and vege-tables intake and frequency of breakfast and snacks consumption: a cross-sectional study. Nutr. J..

[18] Martone D., Roccaldo R., Censi L., Toti E., Catasta G., D’Addesa D., Carletti C. (2013). and ZOOM8 Study Group. Food con-sumption and nutrient intake in Italian school children: results of the ZOOM8 study. Int. J. Food Sci. Nutr..

[19] Censi L., Ruggeri S., Galfo M., Buonocore P., Roccaldo R. (2021). Eating behaviour, physical activity and lifestyle of Italian children during lockdown for COVID-19. Int. J. Food Sci. Nutr..

[20] Kołota A., Głabska D. (2021). Analysis of Food Habits during Pandemic in a Polish Population-Based Sample of Primary School Ad-olescents: Diet and Activity of Youth during COVID-19 (DAY-19) Study. Nutrients.

[21] Hess J.M., Jonnalagadda S.S., Slavin J.L. (2016). What Is a Snack, Why Do We Snack, and How Can We Choose Better Snacks? A Review of the Definitions of Snacking, Motivations to Snack, Contributions to Dietary Intake, and Recommendations for Improvement. Adv. Nutr..

[22] Wang D., Van der Horst K., Jacquier E.F., Afeiche M.C., Eldridge A.L. (2018). Snacking Patterns in Children: A Comparison between Australia, China, Mexico, and the US. Nutrients.

[23] Johnson B.J., Bell L.K., Zarnowiecki D., Rangan A.M., Golley R.K. (2017). Contribution of Discretionary Foods and Drinks to Aus-tralian Children’s Intake of Energy, Saturated Fat, Added Sugars and Salt. Children.

[24] McNicholas J., Hammersley M.L., Hopkins S., McDermott S., Plaskett J. (2022). The Impact of COVID-19 Restrictions on the Healthy Eating and Movement Behaviors of 0–12-Year-Old Children in Western Sydney, Australia. Front. Public Health.

[25] Gage R., Girling-Butcher M., Joe E., Smith M., Ni Mhurchu C., McKerchar C., Puloka V., McLean R., Signal L. (2021). The Fre-quency and Context of Snacking among Children: An Objective Analysis Using Wearable Cameras. Nutrients.

[26] Njike V.Y., Smith T.M., Shuval O., Shuval K., Edshteyn I., Kalantari V., Yaroch A.L. (2016). Snack Food, Satiety, and Weight. Adv. Nutr..

[27] Keast D.R., Nicklas T.A., O’neil C.E. (2010). Snacking is associated with reduced risk of overweight and reduced abdominal obesity in adolescents: National Health and Nutrition Examination Survey (NHANES) 1999–2004. Am. J. Clin. Nutr..

[28] Kerr M.A., Rennie K.L., McCaffrey T.A., Wallace J.M.W., Hannon-Fletcher M.P., Livingstone M.B.E. (2008). Snacking patterns among adolescents: a comparison of type, frequency and portion size between Britain in 1997 and Northern Ireland in 2005. Br. J. Nutr..

