# Supplementary material for: Changes in Food Consumption and Eating Behaviours of Children and Their Families Living in Italy during the COVID-19 Pandemic: The EPaS-ISS Study
Source: Nutrients. 2023 Jul 26;15(15):3326. doi: 10.3390/nu15153326 (PMC10421235; doi:10.3390/nu15153326)
Supplement: Supplementary file 1 [file nutrients-15-03326-s001.zip › nutrients-2528834-supplementary.pdf]

Table S1. Change in children's food consumption between PRE-COVID-19 period and COVID-19 period by socio-demographic characteristics of children and parents.

Foods	Characteristics of children and parents		Decreased (%, 95%CI)	Stayed the same (%, 95%CI)	Increased (%, 95%CI)	Don't Know (%, 95%CI)	*p* value
**Fresh fruit**							
	Child's gender	Female	7.9 (6.82-9.05)	79.8 (78.1-81.5)	7.3 (6.31-8.34)	5.0 (4.15-6.08)	0.7988
		Male	8.2 (7.03-9.45)	80.0 (78.1-81.7)	6.6 (5.67-7.65)	5.3 (4.35-6.38)	
	Geographical areas	North	7.2 (6.17-8.44)	82.2 (80.5-83.8)	6.8 (5.9-7.81)	3.7 (3.0-4.66)	0.0001
		Centre	7.8 (6.31-9.64)	80.6 (78.2-82.8)	6.8 (5.46-8.43)	4.8 (3.63-6.21)	
		South/Islands	9.2 (7.57-11.0)	76.5 (73.8-79.1)	7.1 (5.84-8.66)	7.2 (5.84-8.84)	
	Parents' educational level*	Low educational level	4.7 (2.75-7.9)	77.8 (72.4-82.4)	6.9 (4.37-10.6)	10.6 (7.5-14.9)	0.0000
		Medium educational level	8.2 (7.13-9.51)	80.3 (78.6-81.9)	6.2 (5.21-7.3)	5.3 (4.39-6.39)	
		High educational level	8.2 (7.01-9.66)	81.3 (79.5-82.9)	7.9 (6.76-9.18)	2.6 (2.0-3.39)	
	Parents' nationality	Both Italian parents	7.9 (7.01-8.84)	80.8 (79.5-82.1)	7.2 (6.44-8.05)	4.1 (3.49-4.82)	0.0000
		At least one foreign parent	9.0 (6.98-11.6)	76.4 (72.6-79.9)	5.2 (3.38-7.84)	9.4 (6.96-12.5)	
	Family structure	Two-parent family	8.0 (7.09-8.92)	81.0 (79.6-82.3)	6.9 (6.15-7.75)	4.1 (3.47-4.87)	0.0030
		Single-parent family	8.8 (6.89-11.1)	76.2 (72.5-79.5)	7.4 (5.71-9.68)	7.6 (5.47-10.5)	
**Vegetables**							
	Child's gender	Female	9.3 (8.12-10.7)	80.0 (78.2-81.7)	6.4 (5.57-7.43)	4.2 (3.41-5.14)	0.1755
		Male	8.5 (7.44-9.82)	81.5 (79.8-83.1)	5.2 (4.36-6.18)	4.7 (3.87-5.72)	
	Geographical areas	North	8.7 (7.48-10.1)	81.8 (80.2-83.3)	5.9 (4.97-6.96)	3.6 (2.92-4.52)	0.1047
		Centre	7.8 (6.51-9.31)	81.3 (79.0-83.4)	6.3 (5.03-7.76)	4.6 (3.43-6.22)	

		South/Islands	9.9 (8.12-12.0)	79.3 (76.6-81.7)	5.4 (4.22-6.87)	5.4 (4.34-6.8)	
	Parents' educational level*	Low educational level	7.1 (4.76-10.5)	79.0 (74.4-82.9)	3.7 (1.88-7.05)	10.2 (7.44-13.9)	0.0000
		Medium educational level	10.0 (8.67-11.5)	80.2 (78.3-82.0)	5.2 (4.37-6.23)	4.6 (3.76-5.59)	
		High educational level	8.1 (6.79-9.62)	82.8 (80.9-84.5)	6.6 (5.61-7.87)	2.5 (1.88-3.21)	
	Parents' nationality	Both Italian parents	8.8 (7.8-9.83)	81.9 (80.6-83.1)	5.8 (5.08-6.56)	3.6 (3.05-4.22)	0.0000
		At least one foreign parent	10.1 (7.82-13.0)	75.7 (71.8-79.2)	5.4 (3.61-8.03)	8.8 (6.44-11.8)	
	Family structure	Two-parent family	9.2 (8.29-10.3)	81.3 (79.8-82.6)	5.8 (5.1-6.69)	3.6 (3.07-4.32)	0.0073
		Single-parent family	7.7 (5.78-10.3)	79.6 (76.3-82.6)	6.1 (4.65-8.09)	6.5 (4.81-8.77)	
**Meat**							
	Child's gender	Female	8.6 (7.37-10.0)	81.8 (80.0-83.4)	6.2 (5.27-7.31)	3.4 (2.74-4.24)	0.005
		Male	6.2 (5.33-7.26)	82.2 (80.4-83.8)	8.1 (7.1-9.36)	3.4 (2.7-4.37)	
	Geographical areas	North	7.9 (6.77-9.16)	83.5 (81.8-85.1)	5.8 (4.89-6.8)	2.9 (2.24-3.66)	0.0062
		Centre	7.6 (6.12-9.3)	80.7 (78.0-83.2)	8.3 (6.68-10.3)	3.4 (2.42-4.74)	
		South/Islands	6.6 (5.22-8.22)	80.8 (78.6-82.8)	8.5 (7.3-9.83)	4.2 (3.23-5.37)	
	Parents' educational level*	Low educational level	6.8 (4.62-9.96)	80.4 (75.1-84.8)	6.5 (3.9-10.5)	6.3 (3.87-10.2)	0.0063
		Medium educational level	7.8 (6.65-9.16)	81.4 (79.5-83.2)	7.4 (6.19-8.73)	3.4 (2.73-4.23)	
		High educational level	6.9 (5.8-8.29)	83.5 (81.7-85.2)	7.4 (6.39-8.63)	2.1 (1.56-2.78)	
	Parents' nationality	Both Italian parents	7.1 (6.28-8.01)	82.9 (81.7-84.1)	7.4 (6.64-8.21)	2.6 (2.13-3.1)	0.0000
		At least one foreign parent	9.5 (7.1-12.5)	76.0 (71.8-79.7)	6.6 (4.39-9.85)	7.9 (5.86-10.7)	
	Family structure	Two-parent family	7.4 (6.53-8.3)	82.6 (81.3-83.9)	7.4 (6.59-8.21)	2.6 (2.13-3.23)	0.0193
		Single-parent family	7.4 (5.56-9.67)	81.0 (77.7-84.0)	6.6 (4.75-9.14)	5.0 (3.63-6.81)	

**Fish**							
	Child’s gender	Female	14.9 (13.4-16.6)	76.2 (74.3-78.1)	4.0 (3.16-4.98)	4.9 (4.115.81)	0.2078
		Male	12.9 (11.5-14.4)	78.7 (76.8-80.4)	3.8 (3.1-4.58)	4.7 (3.85-5.7)	
	Geographical areas	North	12.4 (11.1-13.9)	79.8 (77.9-81.5)	4.0 (3.21-4.91)	3.9 (3.13-4.76)	0.005
		Centre	13.5 (11.4-15.8)	77.8 (75.1-80.4)	4.1 (2.91-5.73)	4.6 (3.58-5.9)	
		South/Islands	15.9 (13.8-18.3)	74.4 (71.9-76.8)	3.6 (2.62-4.95)	6.1 (4.94-7.46)	
	Parents’ educational level*	Low educational level	11.7 (8.67-15.5)	76.0 (71.8-79.7)	2.6 (1.39-4.76)	9.8 (6.81-13.9)	0.0000
		Medium educational level	14.2 (12.6-15.9)	77.5 (75.4-79.4)	3.1 (2.4-4.06)	5.2 (4.41-6.25)	
		High educational level	14.2 (12.4-16.2)	78.2 (76.1-80.1)	4.9 (3.98-5.98)	2.7 (2.14-3.52)	
	Parents’ nationality	Both Italian parents	14.7 (13.6-16.0)	77.7 (76.2-79.1)	3.7 (3.13-4.4)	3.9 (3.33-4.55)	0.0000
		At least one foreign parent	7.3 (5.44-9.79)	77.5 (73.4-81.1)	5.1 (3.16-8.12)	10.1 (7.7-13.1)	
	Family structure	Two-parent family	14.4 (13.1-15.7)	77.7 (76.1-79.1)	3.8 (3.2-4.6)	4.1 (3.51-4.89)	0.0827
		Single-parent family	13.6 (11.3-16.3)	75.7 (72.4-78.8)	4.1 (2.7-6.17)	6.5 (4.86-8.78)	
**Dairy products and eggs**							
	Child’s gender	Female	5.7 (4.83-6.75)	82.4 (80.6-84.0)	8.2 (7.08-9.54)	3.7 (2.92-4.67)	0.9499
		Male	5.5 (4.63-6.58)	83.0 (81.3-84.5)	7.8 (6.82-8.96)	3.7 (2.94-4.64)	
	Geographical areas	North	5.0 (4.21-5.95)	83.8 (81.9-85.6)	8.1 (6.91-9.45)	3.1 (2.42-3.86)	0.0385
		Centre	4.9 (3.73-6.37)	84.2 (81.8-86.3)	7.4 (5.95-9.08)	3.6 (2.62-4.92)	
		South/Islands	6.8 (5.42-8.55)	80.3 (78.0-82.5)	8.3 (6.98-9.8)	4.6 (3.45-6.06)	
	Parents’ educational level*	Low educational level	7.1 (4.42-11.2)	78.9 (73.9-83.2)	6.7 (4.49-9.76)	7.3 (4.75-11.1)	0.0007
		Medium educational level	5.4 (4.44-6.45)	82.8 (80.9-84.6)	8.3 (7.18-9.6)	3.5 (2.78-4.44)	
		High educational level	5.4 (4.42-6.66)	84.1 (82.2-85.8)	8.2 (7.04-9.47)	2.3 (1.73-3.12)	

	Parents’ nationality	Both Italian parents	5.5 (4.77-6.31)	83.8 (82.6-85.0)	7.9 (7.11-8.76)	2.8 (2.29-3.4)	0.0000
		At least one foreign parent	5.7 (3.88-8.42)	75.9 (71.6-79.7)	10.1 (7.6-13.3)	8.3 (6.16-11.1)	
	Family structure	Two-parent family	5.6 (4.86-6.44)	83.3 (82.0-84.6)	7.9 (7.08-8.88)	3.1 (2.56-3.8)	0.1410
		Single-parent family	5.5 (3.99-7.44)	80.8 (77.6-83.6)	9.0 (7.08-11.4)	4.8 (3.32-6.85)	
**Pulses**							
	Child’s gender	Female	6.7 (5.85-7.7)	82.0 (80.4-83.4)	5.4 (4.43-6.62)	5.9 (4.96-7.03)	0.6438
		Male	6.0 (5.12-7.09)	83.2 (81.7-84.5)	4.9 (4.04-5.86)	5.9 (5.02-6.95)	
	Geographical areas	North	6.2 (5.34-7.26)	83.3 (81.8-84.7)	4.3 (3.47-5.28)	6.1 (5.19-7.26)	0.1848
		Centre	7.2 (5.85-8.76)	81.7 (79.5-83.8)	5.1 (3.92-6.67)	6.0 (4.84-7.35)	
		South/Islands	6.1 (4.92-7.45)	82.1 (80.2-83.9)	6.2 (4.94-7.85)	5.6 (4.44-6.97)	
	Parents’ educational level*	Low educational level	7.0 (4.65-10.5)	81.4 (76.4-85.5)	2.5 (1.28-4.87)	9.1 (6.2-13.1)	0.0001
		Medium educational level	7.0 (6.01-8.1)	82.3 (80.7-83.8)	4.4 (3.49-5.62)	6.3 (5.32-7.37)	
		High educational level	5.7 (4.68-6.83)	84.1 (82.3-85.7)	6.3 (5.22-7.58)	4.0 (3.16-4.97)	
	Parents’ nationality	Both Italian parents	6.3 (5.58-7.09)	83.5 (82.4-84.6)	5.2 (4.58-6.01)	4.9 (4.32-5.63)	0.0000
		At least one foreign parent	6.9 (5.13-9.33)	78.0 (73.8-81.6)	3.9 (2.42-6.25)	11.2 (8.6-14.4)	
	Family structure	Two-parent family	6.7 (5.94-7.55)	82.9 (81.7-84.1)	5.2 (4.47-6.08)	5.1 (4.48-5.9)	0.0046
		Single-parent family	5.1 (3.68)	81.8 (78.9-84.5)	4.8 (3.59-6.43)	8.3 (6.36-10.7)	
**Savoury snacks**							
	Child’s gender	Female	5.5 (4.54-6.56)	67.7 (65.3-69.9)	23.3 (21.4-25.5)	3.5 (2.8-4.44)	0.5322
		Male	6.3 (5.37-7.33)	66.0 (63.9-68.0)	24.3 (22.6-26.2)	3.4 (2.7-4.3)	
	Geographical areas	North	5.4 (4.47-6.47)	69.7 (67.6-71.8)	21.9 (20.1-23.7)	3.0 (2.39-3.85)	0.0469
		Centre	6.4 (5.1-8.0)	64.8 (61.8-67.8)	25.2 (22.5-28.0)	3.6 (2.61-4.89)	

		South/Islands	6.2 (4.89-7.97)	64.1 (60.7-67.3)	25.7 (22.7-29.0)	4.0 (3.04-5.15)	
	Parents’ educational level*	Low educational level	2.8 (1.46-5.2)	70.5 (65.4-75.0)	20.7 (16.8-25.3)	6.1 (3.72-9.74)	0.0001
		Medium educational level	5.3 (4.4-6.4)	65.1 (62.7-67.5)	26.4 (24.2-28.7)	3.1 (2.46-4.02)	
		High educational level	6.9 (5.73-8.39)	67.7 (65.5-69.8)	22.7 (20.9-24.8)	2.6 (2.01-3.34)	
	Parents’ nationality	Both Italian parents	5.7 (4.97-6.58)	67.2 (65.5-69.0)	24.3 (22.7-25.9)	2.7 (2.29-3.3)	0.0000
		At least one foreign parent	6.9 (4.73-9.93)	62.1 (57.8-66.3)	23.3 (19.4-27.8)	7.6 (5.47-10.5)	
	Family structure	Two-parent family	5.9 (5.10-6.859	67.2 (65.4-69.09)	23.9 (22.3-25.5)	3.01 (2.47-3.65)	0.3468
		Single-parent family	6.1 (4.56-8.07)	65.5 (62.2-68.6)	24.2 (21.3-27.3)	4.3 (3.02-6.03)	
**Sweet foods**							
	Child’s gender	Female	4.5 (3.77-5.47)	66.6 (64.4-68.8)	25.2 (23.2-27.3)	3.6 (2.86-4.53)	0.3373
		Male	5.6 (4.75-6.67)	65.8 (63.7-67.7)	25.5 (23.6-27.4)	3.2 (2.45-4.07)	
	Geographical areas	North	4.8 (3.92-5.76)	68.7 (66.5-70.8)	23.6 (21.7-25.6)	3.0 (2.29-3.89)	0.0194
		Centre	4.3 (3.09-5.83)	66.6 (63.8-69.4)	26.1 (23.3-29.1)	3.0 (2.16-4.09)	
		South/Islands	6.1 (4.82-7.59)	62.7 (59.5-65.8)	27.2 (24.2-30.4)	4.1 (3.08-5.44)	
	Parents’ educational level*	Low educational level	4.7 (2.83-7.71)	65.6 (60.3-70.6)	21.7 (17.7-26.2)	8.0 (5.15-12.3)	0.0000
		Medium educational level	4.9 (3.95-6.0)	65.7 (63.2-68.0)	26.4 (24.1-28.9)	3.04 (2.39-3.87)	
		High educational level	5.4 (4.34-6.71)	66.7 (64.6-68.8)	25.9 (23.9-28.0)	2.0 (1.44-2.66)	
	Parents’ nationality	Both Italian parents	4.9 (4.22-5.66)	66.3 (64.6-68.0)	26.3 (24.7-27.9)	2.5 (2.07-3.08)	0.0000
		At least one foreign parent	6.7 (4.64-9.62)	64.0 (59.5-68.2)	21.5 (17.6-26.0)	7.8 (5.51-11.0)	
	Family structure	Two-parent family	5.1 (4.37-5.85)	66.4 (64.7-68.1)	25.7 (24.1-27.3)	2.8 (2.29-3.48)	0.5961
		Single-parent family	5.4 (3.65-7.95)	65.9 (62.4-69.2)	24.9 (21.5-28.6)	3.8 (2.56-5.75)	

**Packaged drinks containing sugar**							
	Child's gender	Female	4.5 (3.77-5.47)	65.8 (63.7-67.7)	25.5 (23.6-27.4)	3.2 (2.45-4.07)	0.0856
		Male	9.4 (8.3-10.7)	74.7 (72.9-76.5)	11.6 (10.3-13.1)	4.2 (3.45-5.04)	
	Geographical areas	North	8.7 (7.6-10.0)	77.6 (75.7-79.5)	9.6 (8.39-11.1)	4.0 (3.27-4.9)	0.0495
		Centre	10.5 (8.67-12.6)	74.5 (71.3-77.4)	10.9 (9.12-12.9)	4.2 (3.13-5.52)	
		South/Islands	10.9 (9.42-12.6)	72.3 (69.6-74.9)	11.7 (9.56-14.2)	5.1 (4.03-6.36)	
	Parents' educational level*	Low educational level	5.6 (3.53-8.73)	77.2 (72.7-81.2)	9.1 (6.71-12.1)	8.1 (5.55-11.7)	0.0000
		Medium educational level	9.2 (8.0-10.5)	74.9 (72.8-76.8)	11.9 (10.4-13.6)	4.0 (3.26-5.03)	
		High educational level	10.9 (9.6-12.3)	76.1 (74.1-78.0)	9.9 (8.56-11.3)	3.2 (2.51-3.99)	
	Parents' nationality	Both Italian parents	9.4 (8.48-10.5)	76.4 (74.9-77.9)	10.5 (9.41-11.7)	3.6 (3.14-4.25)	0.0000
		At least one foreign parent	12.4 (9.82-15.5)	66.5 (62.0-70.8)	12.9 (10.2-16.1)	8.2 (5.92-11.3)	
	Family structure	Two-parent family	9.7 (8.74-10.8)	76.0 (74.4-77.5)	10.6 (9.53-11.9)	3.6 (3.1-4.31)	0.0039
		Single-parent family	10.5 (8.67-12.6)	72.1 (68.5-75.4)	11.0 (8.81-13.7)	6.4 (4.89-8.46)	
**Breakfast cereals**							
	Child's gender	Female	5.1 (4.23-6.07)	80.9 (79.1-82.6)	5.4 (4.57-6.27)	7.3 (6.17-8.57)	0.0683
		Male	6.3 (5.31-7.43)	81.1 (79.4-82.7)	5.4 (4.57-6.27)	7.3 (6.17-8.57)	
	Geographical areas	North	5.3 (4.39-6.44)	81.7 (80.0-83.2)	6.6 (5.73-7.56)	6.4 (5.41-7.64)	0.1466
		Centre	5.0 (3.73-6.62)	81.3 (78.8-83.5)	6.6 (5.44-8.01)	7.1 (5.74-8.81)	
		South/Islands	6.6 (5.4-8.09)	80.0 (77.6-82.2)	5.3 (4.17-6.73)	8.1 (6.47-10.0)	
	Parents' educational level*	Low educational level	7.2 (4.49-11.3)	77.6 (71.8-82.5)	3.9 (2.38-6.34)	11.3 (7.67-16.4)	0.0020
		Medium educational level	5.8 (4.80-6.90)	80.5 (78.8-82.1)	6.3 (5.37-7.46)	7.4 (6.26-8.66)	

		High educational level	4.8 (3.97-5.75)	83.3 (81.6-84.8)	6.51 (5.46-7.74)	5.4 (4.51-6.54)	
	Parents’ nationality	Both Italian parents	5.4 (4.81-6.17)	82.1 (80.9-83.2)	6.1 (5.43-6.83)	6.4 (5.59-7.29)	0.0038
		At least one foreign parent	6.4 (4.36-9.34)	75.8 (71.6-79.5)	6.7 (4.66-9.63)	11.1 (8.17-14.9)	
	Family structure	Two-parent family	5.5 (4.81-6.29)	81.8 (80.5-83.0)	6.2 (5.52-6.9)	6.5 (5.69-7.51)	0.4000
		Single-parent family	6.4 (4.83-8.51)	79.0 (75.6-82.0)	6.7 (4.92-8.96)	7.9 (5.95-10.4)	

* The highest educational level between the two parents.

Table S2. Change in families’ eating behaviours between PRE-COVID-19 period and COVID-19 period by socio-demographic characteristics of children and parents.

Family consumption behaviours							
			**Decreased (%, 95%CI)**	**Stayed the same (%, 95%CI)**	**Increased (%, 95%CI)**	**Don't Know (%, 95%CI)**	***p* value**
**Buying food online**							
	Child's gender	Female	16.8 (15.2-18.6)	42.2 (39.9-44.5)	18.0 (16.1-20.1)	23.0 (21.0-25.1)	0.4250
		Male	16.9 (15.3-18.7)	40.0 (37.8-42.3)	19.7 (17.8-21.7)	23.4 (21.6-25.3)	
	Geographical areas	North	14.1 (12.6-15.7)	43.5 (41.0-46.0)	25.0 (22.6-27.5)	17.5 (15.7-19.4)	0.0000
		Centre	13.8 (11.8-16.0)	44.0 (40.6-47.4)	17.8 (14.3-21.9)	24.5 (21.6-27.6)	
		South/Islands	22.3 (20.0-24.8)	36.2 (33.3-39.3)	11.6 (9.49-14.0)	29.9 (27.0-32.9)	
	Parents' educational level*	Low educational level	22.5 (18.2-27.5)	31.2 (26.6-36.2)	5.96 (3.73-9.4)	40.4 (35.0-45.9)	0.0000
		Medium educational level	19.5 (17.6-21.6)	36.9 (34.8-39.2)	16.6 (14.8-18.5)	27.0 (24.9-29.2)	
		High educational level	12.9 (11.2-14.9)	47.6 (45.2-50.0)	24.4 (22.0-26.9)	15.1 (13.5-16.8)	
	Parents' nationality	Both Italian parents	16.2 (14.9-17.6)	42.5 (40.7-44.3)	20.3 (18.5-22.1)	21.1 (19.6-22.6)	0.0000
		At least one foreign parent	21.9 (18.0-26.3)	32.8 (28.6-37.3)	10.2 (7.7-13.5)	35.1 (30.6-39.8)	
	Family	Two-parent family	16.6 (15.3-18.1)	41.5 (39.6-43.4)	19.8 (18.0-21.6)	22.1 (20.6-23.7)	0.1052
		Single-parent family	19.4 (16.9-22.2)	40.0 (36.3-43.8)	16.5 (13.6-19.9)	24.1 (20.3-28.3)	
**Buying food in bulk**							
	Child's gender	Female	7.3 (6.26-8.5)	40.8 (38.7-43.0)	46.5 (44.1-48.8)	5.4 (4.5-6.5)	0.2387
		Male	6.0 (5.06-7.13)	40.1 (37.9-42.3)	47.7 (45.4-50.0)	6.2 (5.18-7.41)	

	Geographical areas	North	5.1 (4.28-6.03)	43.3 (41.1-45.5)	46.8 (44.3-49.4)	4.8 (3.81-6.1)	0.0000
		Centre	6.1 (4.88-7.69)	40.2 (37.0-43.6)	48.3 (44.9-51.6)	5.4 (4.2-6.84)	
		South/Islands	8.9 (7.47-10.6)	36.9 (33.7-40.1)	46.9 (43.3-50.5)	7.4 (6.07-8.92)	
	Parents' educational level*	Low educational level	9.5 (6.7-13.2)	48.2 (42.3-54.2)	31.6 (26.2-37.5)	10.7 (7.99-14.2)	0.0000
		Medium educational level	7.1 (6.03-8.36)	41.0 (38.8-43.2)	45.1 (42.8-47.5)	6.8 (5.78-8.03)	
		High educational level	5.1 (4.21-6.06)	38.3 (36.0-40.7)	53.2 (50.9-55.5)	3.4 (2.63-4.37)	
	Parents' nationality	Both Italian parents	6.0 (5.34-6.8)	40.0 (38.3-41.7)	48.8 (47.0-50.7)	5.2 (4.47-5.97)	0.0000
		At least one foreign parent	9.4 (7.31-12.1)	43.6 (39.7-47.6)	37.5 (33.2-42.1)	9.4 (6.72-13.0)	
	Family	Two-parent family	6.2 (5.5-7.09)	39.4 (37.7-41.1)	49.3 (47.5-51.2)	5.0 (4.34-5.82)	0.0001
		Single-parent family	8.1 (6.46-10.2)	44.9 (41.0-48.9)	40.2 (36.3-44.3)	6.7 (5.04-8.99)	
**Eating home-cooked meals**							
	Child's gender	Female	2.6 (1.89-3.55)	53.6 (51.6-55.7)	40.9 (38.9-42.8)	2.9 (2.26-3.75)	0.3591
		Male	2.8 (2.19-3.59)	50.9 (48.6-53.2)	43.4 (41.1-45.7)	2.9 (2.21-3.81)	
	Geographical areas	North	2.6 (1.94-3.44)	54.6 (52.5-56.7)	40.2 (38.0-42.5)	2.6 (1.92-3.51)	0.1237
		Centre	2.6 (1.82-3.82)	51.2 (47.5-54.9)	43.8 (40.3-47.4)	2.3 (1.43-3.66)	
		South/Islands	2.9 (1.98-4.24)	49.7 (46.4-52.9)	43.8 (40.7-46.8)	3.7 (2.73-4.89	
	Parents' educational level*	Low educational level	1.5 (0.67-3.32)	60.9 (54.8-66.7)	31.5 (26.0-37.7)	6.0 (3.59-10.0)	0.0000
		Medium educational level	2.9 (2.22-3.88)	52.7 (50.2-55.1)	41.2 (38.8-43.7)	3.2 (2.47-4.03)	

		High educational level	2.3 (1.79-3.08)	50.3 (47.9-52.7)	46.2 (43.8-48.7)	1.1 (0.732-1.75)	
	Parents' nationality	Both Italian parents	2.4 (1.9-2.99)	51.3 (49.5-53.1)	44.2 (42.4-46.0)	2.1 (1.66-2.66)	0.0000
		At least one foreign parent	3.8 (2.4-6.08)	60.1 (55.7-64.3)	29.7 (25.5-34.3)	6.4 (4.32-9.29)	
	Family	Two-parent family	2.3 (1.88-2.94)	51.5 (49.8-53.3)	43.7 (41.8-45.6)	2.4 (1.92-3.09)	0.0161
		Single-parent family	3.9 (2.6-5.74)	54.4 (50.1-58.6)	38.5 (34.9-42.2)	3.2 (2.09-4.97)	
**Eating food prepared outside the home**							
	Child's gender	Female	27.0 (25.1-28.9)	44.2 (42.0-46.5)	12.9 (11.5-14.4)	15.9 (14.3-17.7)	0.2631
		Male	29.2 (27.3-31.2)	43.7 (41.4-46.0)	12.8 (11.4-14.3)	14.3 (12.7-16.0)	
	Geographical areas	North	25.1 (23.3-27.0)	47.9 (45.7-50.2)	15.9 (14.2-17.8)	11.1 (9.6-12.7)	0.0000
		Centre	25.1 (22.8-27.6)	44.1 (40.8-47.5)	15.1 (13.0-17.5)	15.6 (13.0-18.7)	
		South/Islands	33.8 (31.0-36.8)	38.7 (35.8-41.7)	7.54 (6.2-9.13)	20.0 (17.6-22.6)	
	Parents' educational level*	Low educational level	31.2 (26.5-36.3)	35.7 (30.6-41.1)	5.04 (3.08-8.14)	28.1 (23.8-32.8)	0.0000
		Medium educational level	29.9 (27.9-32.1)	39.9 (37.8-42.1)	12.0 (10.4-13.7)	18.2 (16.4-20.2)	
		High educational level	26.1 (24.1-28.1)	49.5 (47.3-51.8)	15.6 (14.0-17.3)	8.8 (7.6-10.3)	
	Parents' nationality	Both Italian parents	27.7 (26.1-29.4)	44.7 (43.0-46.4)	13.7 (12.5-15.0)	13.9 (12.7-15.0)	0.0000
		At least one foreign parent	32.2 (27.9-36.8)	38.7 (34.2-43.3)	8.01 (5.91-10.8)	21.1 (17.3-25.5)	

	Family	Two-parent family	28.2 (26.6-29.9)	44.7 (42.9-46.5)	12.9 (11.7-14.2)	14.2 (13.0-15.6)	0.1600
		Single-parent family	28.6 (25.6-31.9)	40.9 (37.5-44.3)	13.9 (11.6-16.5)	16.6 (14.0-19.6)	
**Eating together as a family**							
	Child's gender	Female	7.0 (6.01-8.21)	52.5 (50.2-54.7)	38.2 (35.9-40.6)	2.3 (1.65-3.16)	0.1059
		Male	8.2 (6.96-9.73)	49.0 (46.8-51.2)	40.0 (37.7-42.4)	2.4 (2.08-3.47)	
	Geographical areas	North	4.4 (3.61-5.44)	51.5 (49.2-53.7)	42.2 (39.8-44.6)	1.9 (1.36-2.66)	0.0000
		Centre	5.3 (4.23-6.73)	51.2 (47.5-54.9)	41.0 (36.8-45.3	2.5 (1.59-3.78)	
		South/Islands	13.2 (11.1-15.7)	49.3 (45.9-52.7)	34.2 (30.9-37.6)	3.3 (2.42-4.44)	
	Parents' educational level*	Low educational level	12.0 (8.64-16.3)	61.0 (55.2-66.5)	21.9 (16.5-28.5)	5.1 (3.31-7.94)	0.0000
		Medium educational level	8.9 (7.5-10.6)	51.8 (49.4-54.3)	36.5 (34.2-38.9)	2.7 (2.07-3.6)	
		High educational level	5.4 (4.44-6.52)	47.4 (44.8-50.1)	46.1 (43.4-48.9)	1.0 (0.588-1.82)	
	Parents' nationality	Both Italian parents	7.4 (6.37-8.58)	50.0 (48.2-51.8)	40.7 (38.7-42.6)	1.9 (1.52-2.46)	0.0002
		At least one foreign parent	8.0 (5.9-10.8)	55.6 (51.1-60.0)	32.0 (27.5-36.7	4.5 (2.79-7.06)	
	Family	Two-parent family	7.3 (6.3-8.41)	49.1 (47.2-51.0)	41.5 (39.6-43.5)	2.1 (1.63-2.69)	0.0001
		Single-parent family	8.6 (6.66-11.1)	56.9 (53.3-60.5)	31.7 (28.0-35.6)	2.8 (1.77-4.38)	
**Cooking with your child**							
	Child's gender	Female	3.7 (2.92-4.6)	50.1 (48.0-52.2)	42.6 (40.4-44.8)	3.6 (2.9-4.55)	0.2768
		Male	3.9 (3.08-4.83)	49.8 (47.6-52.0)	41.5 (39.1-44.0)	4.8 (3.96-5.76)	

	Geographical areas	North	2.8 (2.21-3.56)	50.9 (48.7-53.2)	42.6 (40.2-45.1)	3.6 (2.79-4.74)	0.0363
		Centre	4.0 (2.9-5.51)	50.7 (47.3-54.0)	41.5 (37.8-45.2)	3.8 (2.71-5.41)	
		South/Islands	4.9 (3.74-6.33)	48.3 (45.2-51.3)	41.6 (37.8-45.5)	5.2 (4.24-6.45)	
	Parents’ educational level*	Low educational level	5.9 (3.82-8.95)	55.6 (50.2-60.8)	29.4 (24.5-34.8)	9.2 (6.75-12.4)	0.0000
		Medium educational level	4.1 (3.32-5.13)	51.2 (49.1-53.4)	39.8 (37.5-42.2)	4.8 (1.45-2.78)	
		High educational level	2.8 (2.16-3.65)	47.1 (44.8-49.4)	48.1 (45.6-50.5)	2.0 (1.45-2.78)	
	Parents’ nationality	Both Italian parents	3.4 (2.79-4.04)	49.2 (47.5-50.9)	44.0 (42.1-45.9)	3.5 (2.96-4.13)	0.0000
		At least one foreign parent	6.8 (4.83-9.52)	54.9 (50.2-59.4)	30.5 (25.6-35.7)	7.9 (5.47-11.2)	
	Family	Two-parent family	3.3 (2.74-4.03)	48.9 (47.2-50.6)	44.0 (42.0-46.0)	3.8 (3.18-4.47)	0.0031
		Single-parent family	5.2 (3.73-7.33)	53.3 (49.9-56.6)	37.1 (33.5-40.9)	4.3 (2.99-6.21)	
		Single-parent family	6.3 (4.69-8.36)	68.6 (65.1-72.0)	16.5 (14.0-19.4)	8.6 (6.57-11.1)	

*The highest educational level between the two parents.

S3. List of the questions considered in the analysis.

Some information about the child

1) What is your relationship to the child?
1. Mother
2. Father
3. Other (please specify): ______________

How old is the child?
(E.g. a child born on 16 September 2013 will be 8 years and 5 months old on 10 March 2022 (date of completion of the questionnaire)
1. Years |__|__|
2. Months |__|__|

The child is
1. Male
2. Months

The next questions concern the impact of the COVID-19 pandemic on daily habits and behaviours

2) Please indicate each month in which the child was mostly prevented from attending school because of the COVID-19 pandemic (e.g. due to national lockdown, being resident in a 'red' zone, being in self-isolation, or because the school attended was closed)

Consider the year 2020 first

2. February	3. March	4. April	5. May	6. June	7. July	8. August
9. September	10. October	11. November	12. December			

Then consider last year, i.e. 2021

1. January	2. February	3. March	4. April	5. May	6. June	7. July
8. August	9. September	10. October	11. November	12. December		

Finally, consider the current year, i.e. 2022

1. January	2. February	3. March	4. April

IMPORTANT: In the following questions, consider the 'PRE-COVID period' as the period before March 2020, before the start of the pandemic (or, in the affected municipalities, before February 2020) and the 'COVID-19 period' as the period in which the child was mostly at home due to the COVID-19 pandemic (see months referred to in the previous question).

3) Please indicate whether there were any changes in your child's consumption of the following foods during the COVID-19 period compared to the PRE-COVID period (less, same, more):

	1. Less than in the PRE-COVID period	2. The same as in the PRE-COVID period	3. More than in the PRE-COVID period	4. I don't know
1. Fresh fruit	☐	☐	☐	☐
2. Vegetables (including soups and pureed vegetables but excluding potatoes)	☐	☐	☐	☐
3. Meat	☐	☐	☐	☐
4. Fish	☐	☐	☐	☐
5. Dairy products (e.g. milk, yoghurt, cheese) and eggs	☐	☐	☐	☐
6. Pulses	☐	☐	☐	☐
7. Savoury snacks (e.g. crisps, popcorn, nuts, crackers)	☐	☐	☐	☐
8. Sweet foods (e.g. cakes, snacks, biscuits, sweets, ice cream)	☐	☐	☐	☐
9. Packaged drinks containing sugar (e.g. tea, orange soda, cola, fruit juices)	☐	☐	☐	☐
10. Breakfast cereals (e.g. corn flakes, muesli)	☐	☐	☐	☐

4) Please indicate which of the following behaviours in a normal week during the COVID-19 period were different from the PRE-COVID period:

	1. Less than in the PRE-COVID period	2. The same as in the PRE-COVID period	3. More than in the PRE-COVID period	4. I don't know
1. Buying regional/local food in local shops or markets	☐	☐	☐	☐
2. Buying food in supermarkets	☐	☐	☐	☐
3. Buying food online	☐	☐	☐	☐
4. Buying food in bulk (over a period longer than a week)	☐	☐	☐	☐
5. Eating home-cooked meals	☐	☐	☐	☐
6. Eating ready meals	☐	☐	☐	☐
7. Eating food prepared outside the home (e.g. takeaway, food ordered online or prepared by a restaurant)	☐	☐	☐	☐
8. Eating together as a family	☐	☐	☐	☐
9. Having breakfast	☐	☐	☐	☐
10. Cooking with your child	☐	☐	☐	☐
11. Using leftover food	☐	☐	☐	☐
12. Planning purchases and meals in advance	☐	☐	☐	☐

5) Questions about the parents:

Mother's nationality
1. Italian
2. Foreign (specify nationality): ___________
3. Mother not present

Mother's level of education
1. None
2. Primary school (ages 6-11)
3. Secondary school (ages 11-14)
4. Secondary school (ages 14-19)

5. Degree
6. Masters/Doctorate/Specialisation

Father’s nationality
1. Italian
2. Foreign (specify nationality): ___________
3. Father not present

Father’s level of education
1. None
2. Primary school (ages 6-11)
3. Secondary school (ages 11-14)
4. Secondary school (ages 14-19)
5. Degree
6. Masters/Doctorate/Specialisation
